# Putting global health high on the agenda of medical schools

**DOI:** 10.1007/s10354-022-00974-7

**Published:** 2022-10-13

**Authors:** Ruth Kutalek, Mina Lahlal, David Kaawa-Mafigiri, Marcella Ryan-Coker, Simone Böll, Sandra Parisi, Phaik Yeong Cheah, Michael Pritsch

**Affiliations:** 1 Medical University of Vienna, Department of Social and Preventive Medicine, Unit Medical Anthropology and Global Health, Kinderspitalgasse 15, 1090 Vienna, Austria; 2 Department of Trauma Surgery, Wilhelminenspital, Vienna, Austria; 3 Department of Social Work and Social Administration, School of Social Sciences, Makerere University, Kampala, Uganda; 4 Department of Surgery, University of Nairobi, Nairobi, Kenya; 5 College of Medicine and Veterinary Medicine, University of Edinburgh, Edinburgh, UK; 6 Department of Dermatology, Inselspital, Bern, Switzerland; 7 Department for General Practice, Universitätsklinikum Würzburg, Würzburg, Germany; 8 Mahidol Oxford Tropical Medicine Research Unit (MORU), Faculty of Tropical Medicine, Mahidol University, Bangkok, Thailand; 9 Centre for Tropical Medicine and Global Health, Nuffield Department of Clinical Medicine, University of Oxford, Oxford, UK; 10 Division of Infectious Diseases and Tropical Medicine, University Hospital, Ludwig-Maximilians-University (LMU) Munich, Munich, Germany

**Keywords:** Global health education, Planetary health, Academic institutionalization, Skills and attitudes, Interdisciplinarity

## Abstract

In this opinion paper, we reflect on global health and global health education as well as challenges that the coming generation are likely to face. As the field is rapidly changing, it is vital to critically reflect categories of “global south” and “global north” as geographical boundaries, and rather think in terms of inequalities that are present in all countries. Global perspectives on health are useful to analyze structural challenges faced in all health care systems and help understand the diversity of cultures and patients’ concepts of disease. We first discuss burning questions and important challenges in the field and how those challenges are tackled. Rather than going into detail on topical issues, we reflect on approaches and attitudes that we think are important in global health education and present opportunities and challenges for young scholars who are interested in working in this field.

## Setting the stage

When asking ten people about their definition of global health, you might receive ten different answers. The field is rapidly changing and evolved from public and international health [1]. Therefore, it is vital to critically reflect categories of “global south” and “global north” as geographical boundaries, and rather think in terms of inequalities that are present in all countries [2]. Global health implies mainly human health, but as it is intriguingly interlinked with animal health and the environment, the related terms of “one health” and “planetary health” have been coined subsequently [3]. Comprehensive approaches to health are important, as human health belongs to a closely interwoven system of many factors and can only be as strong as its weakest part.

In our perspective on global health education, we define global health as the subject of improving health outcomes for vulnerable populations and communities around the globe, using a critical and interdisciplinary approach. Global health aims at leaving no one behind and to therefore diminish or remove health disparities through education, research, and collaborative as well as interdisciplinary action. To us, global health is a principle or a framework to look at health-related challenges and a way to look at health and medicine more comprehensively by including knowledge, methods, theories, and technologies from different disciplines.

Global perspectives on health are useful to analyze structural challenges faced in all health care systems and to help understand the diversity of cultures and patients’ concepts of disease [4]. Understanding social and wider determinants of health and practicing cultural humility is important in all global learning scenarios.

A rapidly increasing number of students want to take courses in the field of global health [5]. For many students, global health and its newer developments are important reasons for taking up their studies as they plan to work in these fields later. While there is an increasing offer of stand-alone global health courses and programs (often with substantial tuition fees) and given the indisputable importance of global health with its skills in general, it is surprising that global health is nearly non-existent in mandatory courses of many medical schools [6, 7]. Academic institutionalization at medical universities or schools is often insufficient and global health seems to be seen more as a personal interest than a profession. Thus, suitable academic programs, positions, and careers are still scarce [6].

In this opinion paper, we reflect on global health and global health education as well as challenges that the coming generation are likely to face. We draw from the expertise and experiences of a diverse set of authors with backgrounds in social sciences, medicine, and global health education. We specifically reflect on the following: What global health concerns will young medical professionals encounter in future? What are important concepts that can be used to tackle those? How can global health be incorporated in education programs? What are opportunities and challenges for young global health scholars?

## Burning questions and important challenges in the field

When talking about burning questions or important challenges in global health, many insecurities exist. However, one thing seems inevitable: Many of the issues can only be approached with interdisciplinarity and solidarity, as they are often intriguingly interlinked and inseparable from each other. It can be difficult and sometimes impossible to quantify their impact and rate them accordingly. Thus, we discuss some critical challenges in the order of our discretion rather than in an evidence-based manner. Consequently, some challenges, although similarly significant, will go unmentioned.

A fundamental challenge of global health is to incorporate an adequate mindset at all times and in all settings, to create sustainable partnerships. As Agnes Binagwaho, pediatrician and former Rwandan Minister of Health, explained: “Before putting physiology and biochemistry in the heads of our students, we need to put global health principles in their hearts” [8]. This can mean finding global solutions to health-related problems or developing local projects with a global perspective.

Some concepts, skills, and attitudes are useful for almost all fields of global health:

- Understanding the mechanisms of poverty and inequality, as well as solidarity and access to adequate health care (including health care of migrants and illegalized people) is fundamental [4].
- A commitment to social and cultural sensitivity and humility is vital as well as the awareness of perceptual filters and biases [9, 10].
- Being able to understand the role of ecological determinants on health, such as resource extraction, deforestation, and mining [11].
- A good understanding of scientific theory can help to understand global health challenges and their larger bio-political implications [12].
- Being able to adapt technologies and reflect on suitable means to create sustainable, accessible, and high-quality health services [13].

Certainly, the following exemplary themes need a coordinated international as well as comprehensive response [14]:

- Climate change with its impact on human, animal, as well as environmental health.
- Antimicrobial drug resistance.
- Stopping infectious diseases and preparing for epidemics, pandemics, or syndemics.
- Caring for and investing in people who care for health.
- Protecting people from potentially harmful products, misinformation, and technologies.
- Management of health in conflicts and crises.
- Mental health in all its forms and other non-communicable diseases.

### Box 1: Antimicrobial drug resistance

Antimicrobial drug resistance (AMR) is already one of the biggest challenges and will be increasingly important in global health. It has been estimated that in 2019, some 4.95 million deaths were associated with and 1.27 million deaths directly attributable to bacterial AMR, with the highest burden in low-resource settings [15]. When taking an economical perspective, the costs of AMR across the globe could account for 100–210 trillion USD by 2050 [16, 17]. AMR and climate change both have enormous costs for society at large [18] and should be an urgent priority for policy-makers and civil society. In the future, we might no longer have effective means of treating severe infections. Moreover, medicine’s great achievements that require effective antimicrobials, such as chemotherapy, major surgery, and organ transplantation, might not be available anymore [19]. Populations who are already worse off, such as those who are living in poverty, in crowded conditions, with poor sanitation, and with poor health care access, will be most impacted by AMR [20]. It is now widely accepted that “the use of antibiotics is the single most important factor leading to antibiotic resistance around the world” [21; p. 11]. Simply using antibiotics creates resistance even when antibiotic use is medically indicated to treat infections. While the driving forces of AMR are biological, the global societal challenge of AMR is the result of human and social practices and values. As an example, large-scale antibiotic use in animal agriculture is connected to nutritional and economic practices and stands for certain values that need to be questioned. Therefore, the approach to any policy to mitigate the problem of AMR needs to be multidisciplinary and include the social, political, and ethical dimensions of AMR.

## Tackling those challenges

The increase in frequency and severity of public health emergencies including AMR or the current COVID-19 pandemic—which by many is considered a syndemic [22] in the way Singer et al. (2017) [23] have coined the term, describing the co-occurrence of two diseases plus social, environmental, and economic factors that worsen disease outcomes—highlights the need to better understand the social and structural contexts of health. A development towards a more critical reflection of those determinants, including an analysis of political decision-making on the EU level and globally, is vital [24]. Likewise, on an academic level, more inclusion, more participation, more diversity, less top-down approaches, and more equal power relations are necessary. This needs to be reflected in the language used [25] and, more importantly, in equal opportunities for researchers in partner countries.

Working in global health requires a constant self-reflective process. Acting against systemic racism in global health as described by Olusanya (2021) [26] is one such process. To achieve this, the new generation of health scientists must focus on reducing inequities and power imbalances in the design and implementation of research. For instance, power dynamics that influence decision-making, e.g., behavior that undermines equitable collaboration in research teams [27] and unequal knowledge transfer process, must be addressed [28]. Capacity-building has to remain a mainstay in the efforts of decolonizing global health. These lessons must be internalized and taught honestly to privileged learners of whom some will work in less privileged settings and communities. Capacity-building must go beyond the acquisition of qualifications and include fair remuneration and rewards such as publication and patenting invention. Scholarly practices and values within collaboration research must reflect the much discussed need to recognize the contribution of all partners [29] in the new global health agenda. Furthermore, whereas power dynamics are fundamentally influenced by funding flow, participatory and community-driven solutions are a must [8]. Moreover, it is essential to recognize and act upon deep-seated and institutionalized gender imbalances in global health. A review of over 200 global organizations active in health showed a so-called 70-80-90 “glass-border” in global health: “70% of leaders in our sample are men, more than 80% are nationals of high-income countries and more than 90% were educated in high-income countries.” It is therefore important to “confront discrimination, inequity and the historical underrepresentation of some groups in the field of global health” [30; p. 10].

## Approaches and attitudes in global health education

**Fig. 1:**
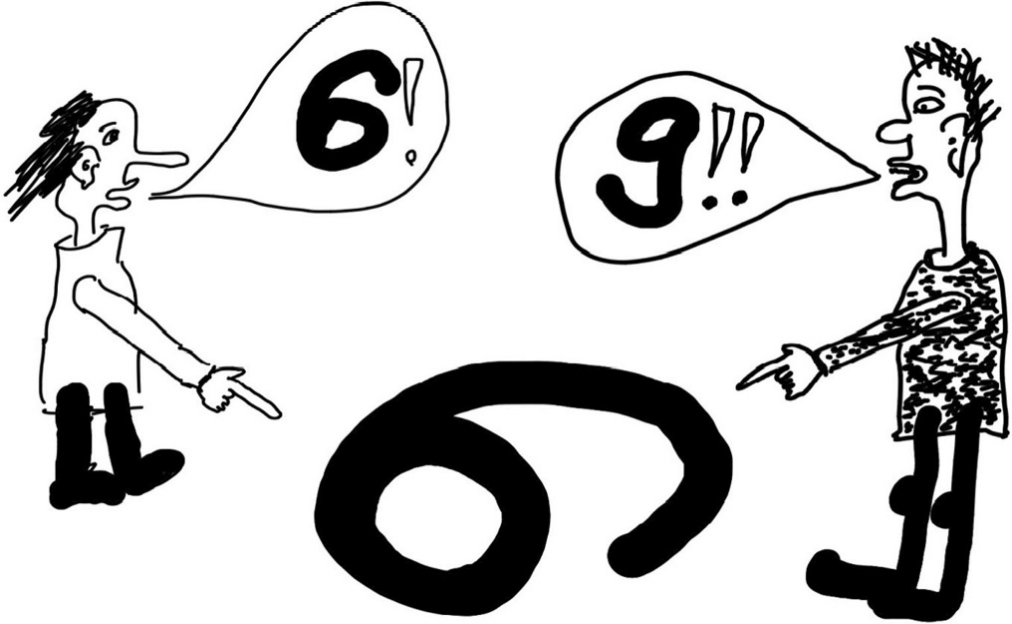
Even if you are completely sure of your truth, trying to understand a conflicting view can help to identify inaccuracies and resolve problems. Sometimes physical travels with associated experiences are very useful or even necessary. (Drawing by M. Pritsch)

All abovementioned aspects should be included in global health education. As an introduction to global health, it seems more suitable to focus on attitudes and approaches using selected, specific examples than on detailed specialized knowledge. As an example, being too sure of one’s opinions and truths can be very harmful and obstruct success, especially in global health. As counterintuitive as it may seem, an important aim thus can be “to confuse” students and promote a feeling of humbleness. Global health challenges seem like kaleidoscopic pictures: If you turn your view slightly, pieces fall together differently, and form a different picture (Fig. 1). Physical travels and mingling with different geographies, climates, mindsets, or cultures leads to valuable insights and promotes adaptability. Those and other suitable tools to identify one’s filters and biases (e.g., journaling, constant self-reflection, studying theories in anthropology, philosophy, psychology) can be laid out to students with an invitation to use and apply them.

When walking a path, it is important to know where you come from and where you want to go. At the moment, we might be evolving from an ego-centric human to a more comprehensive planetary view, generally and in regard to health. This would influence social systems, risk-sharing, or the topic of universal health coverage. Formerly accepted practices like slavery are not accepted anymore (although still practiced while naming it differently). In future, this might also be true for the misuse of animals and the maltreatment of the environment. Studies on the microbiome and viriome of the human body or on how environmental and animal health are interlinked with human health point us to the understanding that humans and their bodies seem more like ecosystems, not as individualistic and separate as once considered. Moreover, there is a strong link between social equity and microbial exposure, as social groups are exposed to microbes in different ways [31].

The digital revolution might additionally change the course of humanity: Provocatively expressed, technology might soon be superior to humans. This could direct us towards more ecological humility and some important questions: What makes us humans human? What right of existence do humans have, also in relation to animals, plants, and the environment? Some truths or concepts are difficult to grasp for human brains. Hypercomplexity is one example: Small changes can result in large and unexpected consequences, often without even noticing them or being able to establish cause and effect. Other examples are the calculation of risks which are sometimes difficult to grasp and to communicate, or fundamental questions of life, e.g., on the origin and end of consciousness. All those mentioned aspects and many more seem important, and students can be stimulated to contemplate on them—generally and by using specific case studies from the field of global health.

## Opportunities and challenges for young scholars

**Fig. 2:**
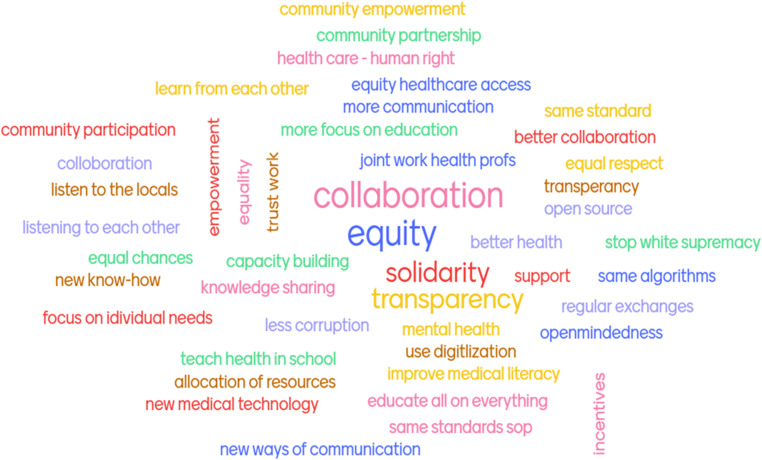
Answers to the mentimeter question “Global health/surgery: What do you want for the future? What do colleagues from lower-resource settings want?” Sample: 42 students from the course “Global Health and Humanitarian Work” at the Medical University of Vienna. All participants gave at least one answer, which are depicted in a word-cloud format. Answers that were mentioned more frequently are increased in size

Students often have a very good sensorium and understanding of the core issues of global health. When we asked students of our 2021 course “Global Health and Humanitarian Work” what they wished for the future of global health, a total of 42 participants answered “collaboration” and “equity” (Fig. 2).

As mentioned before, global health education and activism represent opportunities for transformative learning: Expanding one’s horizon, developing language skills and cultural sensitivity, uncovering blind spots, and personally experiencing the various aspects of the existing and arising global health challenges. Immersion and active involvement in this field can influence attitudes and sensitivity in future medical practice and shape career choices [32]. For example, medical school graduates in the US who participated in global health electives during their studies were more likely to engage in careers with underserved populations [33]. Global health education also leads to favorable attitudes towards marginalized local patient populations [34]. International students’ organizations have been shown to be innovators, filling the gaps in fields such as global surgery [35] or publishing global health performance metrics for universities in the USA, UK, Germany, and Canada [36]. However, there are few global health education programs in other regions, e.g., Asia, Oceania, or South America [37, 38]. Moreover, when it comes to research opportunities, scholars and researchers from low-resource settings face many constraints: Publication fees, inequitable research partnerships with researchers of high-resource settings, discordance in research priorities, lack of funding for research relevant to their context [39]. These ingrained prejudicial practices should be dismantled, while resource-sharing capacities should be strengthened. We need fair, reciprocal, and equitable partnerships for young scholars regardless of their resources. For global surgery, hospital work needs flexibility and, in order for global health and its branches to unfold and reach their full potential, time and energy are required. This mostly exceeds an out-of-hours “leisure” commitment and, as a result, dedicated residency programs have emerged [40].

A significant percentage of medical students spend part of their studies abroad and want to include these experiences into their training [41]. And while global mobility has been pushed through the European Erasmus program and International Federation of Medical Students’ Associations programs, global health education is still lacking in the medical curricula [42]. Instead of singular short-term international experiences that can lead to frustration, exploitation, unintended negative consequences among local communities, traumatization, and the so-called “voluntourism,” long-term educational opportunities and creation of networks that nurture transformative and sustainable learning are needed. Adequate preparation and debriefing increase self-reflectivity and sensitize students for the importance of “first do no harm” [43].

Lack of mobility, language barriers, and unequal exchanges can limit one’s professional career and clinical practice. While global health electives in resource-poor countries frequently offer a lot of clinical possibilities for visiting medical trainees—with all the ethical problems this may involve [44]—visiting doctors from outside the European Union who want to work in the EU have been withdrawn the right to act clinically under supervision in some European countries, and have thus been reduced to mere bystanders. As an example, the regulations for non-EU guest doctors in Austria are very restrictive and bureaucratic and often only allow an “observership” without being able to interact with patients [45]. This lack of reciprocity has made it increasingly difficult for colleagues of resource-poor countries to be actively involved in clinical work and it has also led to the seize of longstanding cooperation programs, e.g., with shared surgical teaching in Cape Verde and Austria [46]. In a globalized medical and surgical world, such obstacles to establishing shared skills and networks have to be considered outdated, reactionary, and as hampering patient care on a worldwide scale.

## The way forward

All in all, global health learning represents an opportunity for shaping the change-makers of the future and vice versa. It can rely on divergent learning, global education techniques, and artistic measures such as graphic medicine (which uses graphic novels) to translate complexities [47, 48]. Aspiring professionals should be aware of existing innovation hubs in low-resource settings, e.g., for surgery (in India [49]), for providing care (e.g., multidisciplinary centers for sexualized violence in Rwanda [50]), for open-source multilingual journals with no paywall [51], or for large-scale cooperations including low-resource settings in residency training (e.g. Pan-African center for neurosurgery in Morocco [52]). The future of the new generation of scholars in global health relies on access to resources, movement, knowledge, skills, quality training and capacity-building, and forming equitable partnerships. They must be empowered with the skills and knowledge necessary to sustain global health. The main goal of this opinion paper was to stimulate thoughts and discussions as well as to give some specific examples, with the ultimate goal to promote education in global health to be part of core curricula for medical students.
